# Exploring Acylcarnitine Metabolism Using Reverse Metabolomics

**DOI:** 10.1021/acs.analchem.6c01418

**Published:** 2026-06-04

**Authors:** Allison J. Keys, Daniel J. Wiener, Sara M. Pacini, Chengze Sun, Lindsay E. Sandusky, Huaiyang Zhong, Emily C. Gentry

**Affiliations:** † 1757 Virginia Tech, Department of Chemistry, Blacksburg, Virginia 24061, United States; ‡ Virginia Tech, Grado Department of Industrial and Systems Engineering, Blacksburg, Virginia 24061, United States; § Hong Kong University of Science and Technology (HKUST), Department of Electrical Engineering, Kowloon, Hong Kong 999077, China

## Abstract

Untargeted mass spectrometry (MS) is a valuable tool for studying human metabolism and identifying small molecule disease biomarkers. However, annotation of chemical structures and validation of findings across numerous cohorts remains challenging. Reverse metabolomics employs a structure-driven approach to overcome these issues by searching spectra of known structures against an entire repository of untargeted LC-MS/MS data to see where metabolites of interest are found. This work uses reverse metabolomics to study acylcarnitine (AC) metabolism in humans and other animals. Here, a library of 76 ACs was chemically synthesized then searched against public metabolomics data to explore where metabolites of interest are detected. From this analysis, it was determined that acylcarnitines are most frequently observed in human and mouse samples, with about 90% of all searched AC structures present in both blood and fecal samples from these species. This work identified positive associations between certain AC structures and disease, indicating their capacity as health biomarkers. Machine learning was applied, determining that AC presence and absence data can accurately predict healthy versus unhealthy individuals with good precision and recall, albeit the models lack disease specificity. Overall, our findings suggest that AC profiles can serve as valuable biomarkers for disease detection throughout the entire lifespan and should be examined for their potential beyond current clinical screening protocols.

## Graphical abstract

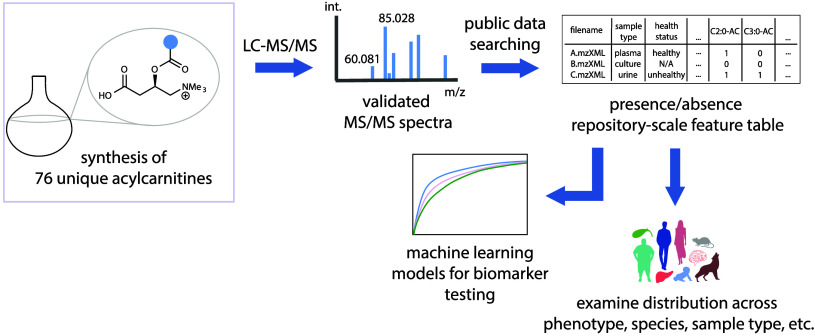

## Introduction

Acylcarnitines (ACs) are critical metabolites to humans, primarily responsible for the transportation of fatty acids across mitochondrial membranes in β-oxidation.^1^ These compounds are routinely analyzed in blood samples of newborns to screen for genetic fatty acid metabolism disorders, but their levels are typically not tracked beyond early life.^2–4^ Extensive work over the last 60 years has studied the chemistry and biology of ACs, demonstrating that circulating levels vary with age, diet and lifestyle,^5–10^ and that altered AC profiles are associated with a range of chronic inflammatory conditions, including atherosclerosis, diabetes and Alzheimer’s disease.^11–13^

Collectively, these findings highlight the potential value of ACs as biomarkers throughout our entire lifespans, yet clinical testing of ACs remains rare in adults, typically restricted to patients with unexplained myopathies or metabolic dysfunction.^14^ Recent advances in liquid chromatography-tandem mass spectrometry (LC-MS/MS) and informatic workflows have improved annotation and quantification of ACs in human blood and urine, expanding opportunities for comprehensive profiling.^15–20^ Distinct fragmentation patterns have been identified for ACs and commonly leveraged to detect their structures across samples, tissues and data sets. For example, 157 different AC masses were identified in six NIST urine samples using four characteristic fragment ions. In another study performed across two human sample types and five different mouse tissues, 514 unique acylcarnitines were quantified from 45 commercial standards using predicted fragmentation patterns and retention times. However, identification of robust AC-disease associations remains challenging and reports in matrices other than blood and urine remain rare.^21–26^ This work synergizes organic synthesis, mass spectrometry and public metabolomics data mining to vanquish these limitations, searching MS/MS spectra of ACs across >900,000 public LC-MS/MS files in the Global Natural Products Social molecular networking (GNPS2)/MassIVE repository, to determine their presence across many clinical cohorts and sample matrices.^27^

To systematically examine AC metabolism at the repository scale, we report a synthesis-basedreverse metabolomics analysis of ACs, where MS/MS spectra from 76 unique synthesized ACs across four structural classes are searched against public metabolomics data (Figure 1a). Reverse metabolomics is a structure-driven workflow that identifies molecules of interest first, then leverages the MAss Spectrometry Search Tool (MASST) and PanReDU platform to explore where these compounds are detected in biological systems.^28–32^ Similar to untargeted metabolomics, reverse metabolomics is well suited for hypothesis generation and biomarker discovery.^33^ However, it takes a more focused approach by targeting specific compound classes rather than the entire metabolome, thereby avoiding a major bottleneck of untargeted workflows: structural annotation of MS/MS spectra. While it does not provide quantitation like targeted metabolomics, it eliminates the need for a priori metabolite selection before data acquisition. In addition, reverse metabolomics enables qualitative meta-analysis at the repository scale, integrating multiple cohorts to improve the robustness of findings. Here, we use synthesis-based reverse metabolomics to determine the prevalence of ACs in different tissues and biofluids, and examine their utility as metabolic markers for human health outcomes.

**Figure 1:**
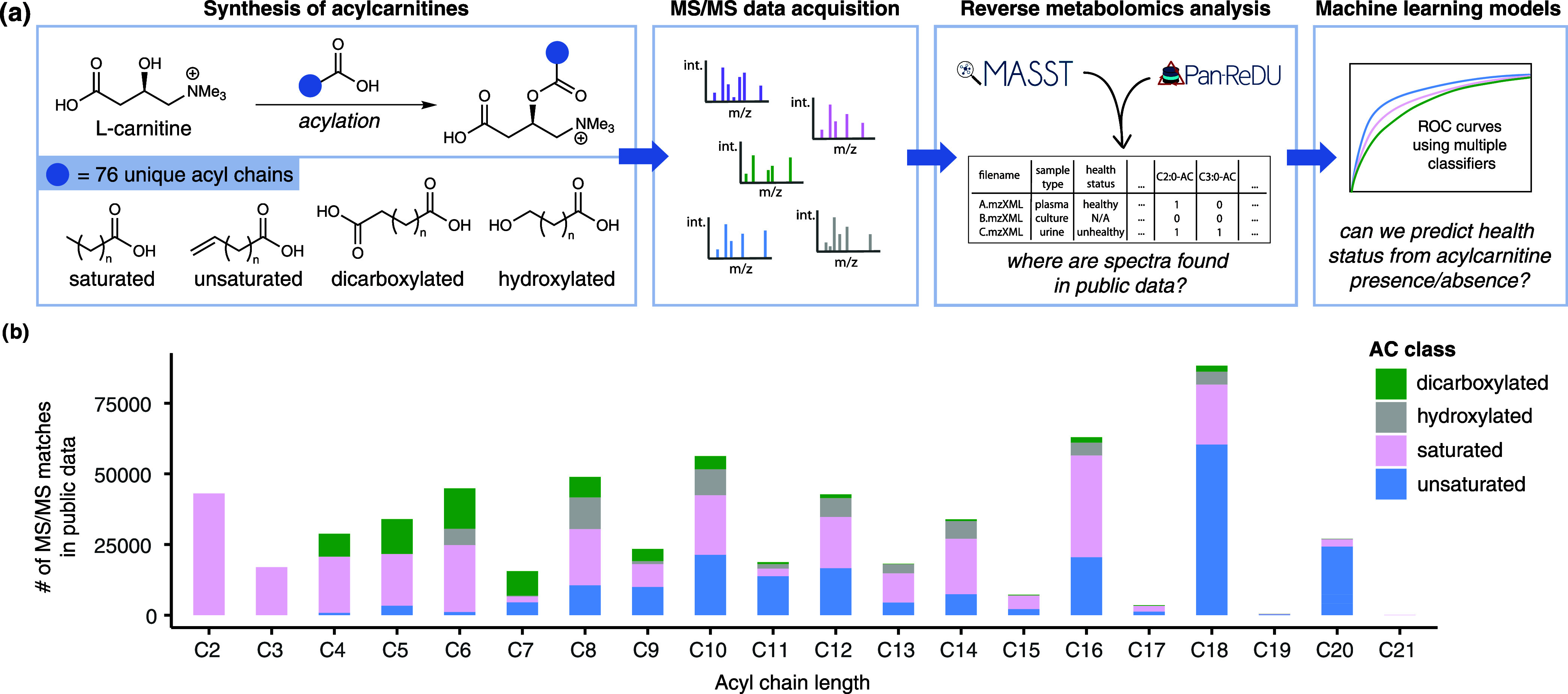
Overview of reverse metabolomics workflow for repository-scale detection of acylcarnitines (ACs) and initial results from analysis. (a) In our reverse metabolomics approach, saturated, unsaturated, dicarboxylated and hydroxylated ACs were synthesized then their MS/MS spectra were searched against public metabolomics data. Machine learning modeling was performed to assess how well the presence and absence of ACs predicts health outcomes. (b) The number of MS/MS matches to unique public data files are shown across fatty chain lengths of ACs.

## Methods

### Synthesis of Acylcarnitines

A library of 76 ACs with unique molecular formulas was assembled, where fatty acyl chains ranged in length from C2 to C21. 1-ethyl-3-(3-(dimethylamino)­propyl)­carbodiimide (EDC)-mediated coupling reactions were used to synthesize saturated, unsaturated, dicarboxylated and hydroxylated ACs from L-carnitine and their corresponding fatty acids (Table S1). Each fatty acid was activated with 4-dimethylaminopyridine (DMAP) and EDC·HCl in dimethylformamide (DMF) and allowed to stir for 15 min before carnitine was added in a single portion at room temperature. Reaction mixtures were allowed to stir overnight at room temperature, then small aliquots were dried and stored at −20 °C until LC-MS/MS data acquisition. For hydroxylated ACs, the fatty acid alcohols were protected with TBSCl prior to acylation reactions. More details on synthetic protocols are provided in Supporting data S1.

The panel of 76 acylcarnitines (ACs) was selected based on two considerations: (i) the availability of corresponding fatty acid precursors for chemical synthesis and (ii) reported prevalence in human metabolomics data sets. Specifically, the selected compounds encompass the most commonly detected short-, medium-, and long-chain saturated and unsaturated ACs in prior comprehensive AC profiling studies in mammalian biofluids and tissues, as well as structurally representative dicarboxylated and hydroxylated species.^15–18,34,35^ Overall, our searches cover 68% of AC structures reported in LIPIDMAPS.^36^ Structures not included in this manuscript either did not perform well in synthesis (e.g., DC2:0) or require prohibitively expensive fatty acid precursors (e.g., hydroxyoctadecatrienoylcarnitine). While not exhaustive, this panel was designed to systematically capture the most ubiquitous ACs encountered in human metabolomics data sets.

### LC-MS/MS Data Acquisition and Processing

For LC-MS/MS analysis, synthetic mixtures were diluted to a final concentration of 40 μM in 50% MeOH/H_2_O solution. Chromatographic separation was performed on a polar C18 column (Kinetex Polar C18, 100 × 2.1 mm^2^, 2,6 μm particle size, 100 Å pore size, Phenomenex) using a Vanquish UHPLC system coupled to a Exploris 120 Orbitrap mass spectrometer (Thermo Fisher Scientific). The mobile phase consisted of solvent A (water +0.1% formic acid) and solvent B (ACN + 0.1% formic acid) and the column compartment was kept at 40 °C. Five microliters were injected for each sample and eluted at a flow rate of 0.5 mL/min using the following general gradient: 0–1.5 min 5% B, 1.5–7.0 min 30% B, 7.0–8.5 min 100% B, 8.5–10.0 min 100% B, 10.0–10.1 min 5% B, 10.1–11.5 min 5% B. A different gradient was used for the C18–C21 hydroxylated acylcarnitines, and details can be found in Supporting data S2.

Mass spectrometry (MS) analysis was performed using heated electrospray ionization (HESI) in positive ionization mode. The parameters were set as follows: Sheath gas to 50, auxiliary gas at 10, sweep gas at 2, spray voltage to 3.5 kV, ion transfer tube to 350 °C and vaporizer to 150 °C. An MS scan range from *m*/*z* 100–1500 was used with an expected peak width of 6s and resolution of 60,000 with 1 microscan. The automatic gain control (AGC) target was set to 1E6 with a maximum injection time of 100 ms. A targeted mass inclusion list was provided and dependent scans were performed on up to the 4 most intense ions per MS1 if no target was detected. MS/MS were acquired with a resolution set to 15,000 with 1 microscan, a maximum injection time of 100 ms and an AGC target of 1E5. The isolation window was set to 2 *m*/*z* and the isolation offset was turned off. Collision energies between 35 and 45 eV were used and selected so that the precursor ion for each compound is approximately 10–20% relative intensity, ensuring complete fragmentation without losing information about the precursor ion. Collision energies used for collection of each MS/MS spectra are listed in Table S1.

Raw LC-MS/MS files were converted to the.mzML format with MSConvert (ProteoWizard, Palo Alto, CA) and uploaded to GNPS2 for subsequent data processing.^27,37,38^ Using the MS/MS-Chooser workflow, spectra for the [M]^+^ ion of all desired products were extracted from the.mzML files using molecular SMILES inputs.^39^ This process also designated each MS/MS with a Unique Spectrum Identifier (USI) output (Table S1), which was used in subsequent searching.^40^

### Quality Control of MS/MS

Establishing the quality of input spectra is essential to the reverse metabolomics process. To ensure crude samples provided the same quality spectra as pure standards, MS/MS spectra were collected for commercially purchased C2:0, C3:0 and C4:0 ACs and compared to those from the crude reaction mixtures (Figure S1 and Table S3). These ACs were chosen because they are highly polar and coelute with residual starting materials and reaction byproducts, which makes their MS/MS acquisition most susceptible to interfering chimeric spectra. In these examples, MS/MS spectra and search results between crude vs pure products were indistinguishable with cosine similarity values greater than 0.999. Based on these findings, MS/MS spectra were acquired from crude samples for the remaining structures. However, before being searched against public LC-MS/MS data, each MS/MS spectra was manually inspected for quality, ensuring correct fragmentation and high spectral purity (Figure S2).

The effects of regio- and stereoisomerism of fatty acyl chains on MS/MS spectral matching were also examined. MS/MS spectra and MASST search results were compared for four isomeric C18 monounsaturated ACs made from petroselinic, oleic, elaidic and vaccenic acids and two isomeric C18:2-derived structures made from linoleic and linolelaidic acid. To determine how the position of the alcohol group affects fragmentation for hydroxylated ACs, two octanoic acid-derived ACs with hydroxyl groups at the C3 and C8 positions were also examined. In all cases, MS/MS spectra of regio- and stereoisomers were nearly identical to one another with cosine values greater than or equal to 0.997 (Figure S3). More information can be found in Supporting data S2 and Table S4. Therefore, the regio- and stereochemistry of acyl groups used in this study were based on availability and cost of fatty acid starting materials. For hydroxylated ACs, terminal fatty acids were also preferred because they lacked an additional stereocenter to consider (Figure S4).

### Reverse Metabolomics Analysis

After passing quality control, MS/MS spectra of the synthesized ACs were searched against public metabolomics data files in the GNPS/MassIVE database.^27^ Specifically, USIs of the verified MS/MS spectra were input into fast MASST (FASST), searching the fragmentation spectra against all public data in GNPS2.^31^ To minimize false discovery rates, the minimum cosine similarity score for a match was set to 0.8 with ion tolerances of 0.02 Da and four minimum matching ions.^41^ Acetylcarnitine was filtered to a minimum cosine of 0.9 and a minimum matched fragment ion of 3, since it had a low number of fragment ions. All searches were performed using *metabolomicspanrepo_index_latest* in late January 2026. These searches give a taskID for each FASST Batch workflow, which can be found in Table S1, along with the USIs for each acylcarnitine used in the study. Results from FASST searches were compiled, removing the scan numbers and duplicate files, to provide lists of unique file matches for each acylcarnitine. This data was combined into a binary feature table with presence/absence data, showing which acylcarnitines were detected in each unique public data file (Table S2). Metadata from PanReDU (Reanalysis of Data User Interface), downloaded on January 26th, 2026, was included in analysis when possible to gain insight on how AC structures are distributed across metadata categories like sample type and species.^30,32^ A detailed breakdown of the phenotypic information for data files in GNPS2 and PanReDU can be found in Table S6. Results were visualized using R and all code used to produce figures can be found on GitHub (https://github.com/keysaj11/Acylcarnitine-Paper).

### Reanalysis of Public Data Sets

Two publicly available untargeted LC-MS/MS data sets from humans were reanalyzed for relative quantitation of ACs. MSV000084556 is a public data set in GNPS/MassIVE that follows individuals on an anti-inflammatory dietary intervention for rheumatoid arthritis. ST002075 is a public data set on Metabolomics Workbench that compares stool metabolomes with those captured by pill capsule endoscopy. For MSV000084556, MS1 feature detection and MS/MS pairing were performed from the.mzML data files using MZmine3.^42^ An intensity threshold of 2E4 and 5E2 were set for MS1 and MS2 detection, respectively, with centroid data. MS1 chromatogram construction was performed using the ADAP chromatogram builder, where the minimum group size was set to 5, group intensity threshold was 2E4, minimum highest intensity was 6E4 and mass tolerance was 0.005 *m*/*z* or 10 ppm. Chromatogram deconvolution was performed using a local minimum search algorithm with a chromatographic threshold of 95%, a search minimum in retention time (RT) range of 0.2 min, minimum relative height of 1%, minimum absolute threshold height of 6E4, minimum ratio for top/edge of 1.05 and a peak duration of 0.01–2 min. Pairing between MS1 and MS2 was performed with a mass tolerance of 0.005 *m*/*z* or 10 ppm and RT range of 0.2 min. Isotope peaks were grouped, then features from different samples were aligned using the same mass and RT tolerances; alignment was performed by placing a weight of 75 on *m*/*z* and 25 on RT. Gap filling was performed with an intensity tolerance of 25%, an *m*/*z* tolerance of 0.005 Da or 10 ppm and a retention time tolerance of 0.2 min. A peak area feature table was exported as a.csv file and consensus MS/MS spectral data were exported in.mgf format. Feature-based molecular networking was performed, which can be accessed from this link: https://gnps2.org/status?task=6562acc9c954432db1f920f26bbef79c. Using MS/MS matching, ACs were identified and peak areas were plotted with R. Peak area abundances for ST002075 were readily available for download from Metabolomics Workbench and plotted using R.

### Building and Testing Machine Learning Models

Using the presence–absence feature table compiled from reverse metabolomics analysis (Table S2) with health status available, we trained supervised machine-learning models to predict sample health status from acylcarnitine detection patterns across public LC-MS/MS files. Each row in the feature table corresponds to a unique GNPS/MassIVE data file and each column corresponds to one synthesized acylcarnitine, encoded as a binary indicator of whether that acylcarnitine was detected (1) or not detected (0) in that file. Outcome labels were derived from the PanReDU health-status ontology, restricting to four mutually exclusive categories: acute illness, chronic illness, healthy and unhealthy (NOS). Files without a health-status annotation were excluded from model development. We used a stratified train/test split (80/20) to preserve class proportions; the held-out test set contained 780 samples, and all reported metrics were computed on this independent test set.

We benchmarked a suite of linear and tree-based classifiers, including multinomial logistic regression with L1 and L2 regularization, a linear support-vector machine, and Naïve Bayes models suited for binary inputs (Bernoulli and multinomial variants). We additionally trained ensemble models (Random Forest and Gradient Boosting) to capture nonlinear interactions among acylcarnitine detections. For linear models, features were standardized where appropriate; for all models, hyperparameters were selected on the training set using cross-validation, and final model performance was evaluated on the fixed test set.

Model performance was summarized using accuracy, macro- and weighted-F1, macro/micro AUROC, and macro/micro AUPRC, along with per-class F1 scores to assess performance across health-status categories (Table S5). To improve interpretability, we used SHAP (SHapley Additive exPlanations) values for the tree-based models to quantify the contribution of individual acylcarnitine features (and metadata covariates when included) to predicted class probabilities, reporting global feature importance and class-specific beeswarm plots. Across the evaluated models, tree-based ensembles (Random Forest and Gradient Boosting) provided the strongest overall discrimination among the four health-status classes, and inclusion of LifeStage and BiologicalSex covariates improved performance relative to acylcarnitine features alone.

### Limitations

In this study, MS/MS spectra of regio- and stereoisomers cannot effectively and reliably be distinguished in MS/MS matching (Figures S1 and S3). Therefore, spectral matches should not be interpreted as the detection of completely identical molecules, but rather detection of all related structures that share fragmentation patterns. Absolute stereo- and regio-chemistry can be determined in downstream validation with complementary techniques and biosynthetic logic. Additionally, the methods used in this paper rely on the reuse of publicly available data, which brings variability into collection and reporting. LC-MS/MS data sets used in repository-scale searches were collected using different instrument platforms and parameters. However, previous work has shown that results from MS/MS matching are similar when spectra are collected on different platforms.^28^ Analysis with metadata was limited to that which is publicly available on PanReDU and most data files with matches do not have associated sample information. Additionally, Machine learning models were exclusively trained and tested on data in PanReDU. Therefore, the generalizability of this model on independent cohorts remains uncertain, so validation in independent cohorts is a priority for subsequent studies.

## Results and Discussion

Acylcarnitines with 76 unique molecular formulas were synthesized, analyzed by LC-MS/MS, then tandem mass spectra for the [M]^+^ ions were extracted and searched for in public metabolomics data. Overall, the searched ACs were detected in 103,243 unique data files across 1,204 data sets deposited in the GNPS/MassIVE database and all synthesized acylcarnitines were present in at least one public datafile (Figure 1b). Even-numbered fatty acyl chains were detected markedly more frequently than odd chain lengths, present in 96,097 and 55,118 data files, respectively. Chain lengths of C2, C16 and C18 were most frequently detected in public data. Of all data files containing ACs, around 34% (37,690 samples from 464 data sets) had entries in the PanReDU database, which provides additional information about sample origin using defined ontologies. Leveraging this metadata, we determined that 86% of all MS/MS matches to AC spectra were from mammals, the majority of which were from humans (18,759 data files) or rats and mice (12,510 data files) (Figure 2a and Table S6). Other animal classes including insects, birds and fish also contained some AC spectra, but this accounted for just 5% of matches. As expected, ACs were rarely detected in fungal or bacterial cultures and environmental samples (Figure S5). A complementary Mass Spectrometry Query Language (MassQL)-based reverse metabolomics approach, reported during revision, yielded consistent observations and greatly expanded structural knowledge of carnitine metabolism using diagnostic ion filtering strategies.^43^

**Figure 2:**
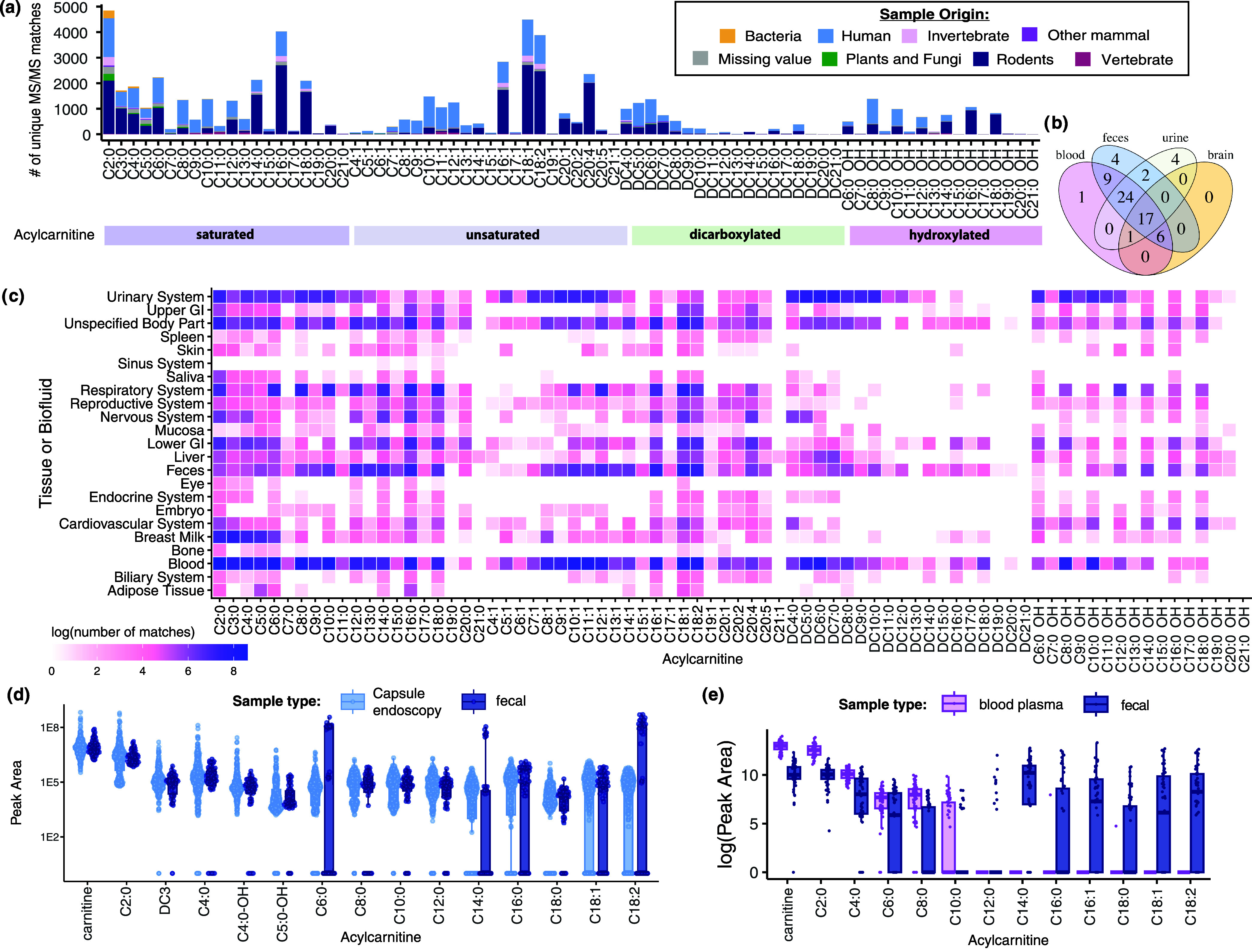
Overview of MASST results showing distribution by sample origin and sample type available in PanReDU. (a) Stacked bar graph displaying the number of unique MS/MS matches per acylcarnitine in PanReDU by sample origin. (b) Venn diagram showing the number of unique AC structures detected in blood, fecal, urine and brain samples. (c) Heatmap displaying the number of MS/MS matches on a logarithmic scale for acylcarnitines across tissues and biofluids. (d) Peak area abundances for acylcarnitines in blood plasma (*n* = 60) and fecal (*n* = 51) samples from MSV000084556. (e) Log of peak area abundances of acylcarnitines in fecal sample (*n* = 57) vs pill capsule endoscopy samples (*n* = 274) from Metabolomics Workbench study ST002075.

Since MS/MS matches for ACs were primarily found in humans, mice and rats, the distribution of ACs across biofluid and tissue samples was explored using this subset of data. Our analysis revealed that distribution of ACs in the body depends on fatty acyl chain identity. Urine samples capture short- and medium-chain acylcarnitines while blood and fecal samples provide a wide range of structure coverage, spanning short, medium and long acyl lengths (Figure 2b,2c). ACs with even-numbered chain lengths were detected at higher rates, but some sample types contributed to this trend more than others. Respiratory samples contained mostly ACs with even-numbered acyl groups, whereas fecal and blood samples had more balanced frequencies of even versus odd chain lengths. Hydroxylated ACs were disproportionately observed as even-chain species across all tissues and biofluids.

Taken together, our results are consistent with established features of fatty acid metabolism, in which even-chain lipids predominate.^44–46^ Mammalian biosynthesis primarily yields even-chain fatty acids, whereas microorganisms generate both odd- and even-chain acyl structures.^47,48^ Accordingly, in our analysis, fecal samples contain both even- and odd-chain acylcarnitines (ACs), with odd-chain species likely reflecting contributions from gut microbiota and dietary intake (e.g., dairy and fermented foods). The presence of these metabolites across many matrices suggests transport into circulation, followed by excretion or distribution to peripheral tissues. Consistent with the enrichment of even-chain ACs observed in respiratory samples, prior studies have reported accumulation of specific even-chain ACs in the lungs.^49,50^ In addition, hydroxylated ACs were detected predominantly as even-chain species, which is consistent with limited selectivity between odd- and even-chain substrates by cytochrome P450 hydroxylases.^51^

From this work, it is clear that ACs are as ubiquitous in fecal samples as they are in blood or urine. ACs have been frequently examined in blood and urine matrices, but they are less frequently reported in stool. Interested in the significant presence of ACs in gut-related samples, we reanalyzed two publicly available untargeted LC-MS/MS data sets from human cohorts. First, to compare relative AC levels in feces vs blood, peak areas were plotted for blood and fecal samples from MSV000084556, a public data set from individuals on dietary intervention for rheumatoid arthritis.^52^ A greater number of structures were detected in feces compared to blood with longer chain structures exclusively detected in fecal samples (Figure 2d). Next, we examined relative abundance of ACs in samples taken during pill endoscopy versus fecal collection in Metabolomics Workbench data set ST002075 to determine whether fecal profiles accurately reflect the gut AC pool.^53^ Peak areas were found to be similar between sample types, validating the prevalence of ACs in the gut (Figure 2e).

Acylcarnitines have been measured extensively in metabolomics experiments to correlate their levels with health outcomes. However, results are often derived from a single data set rather than an entire database, and can lack robustness when compared across cohorts due to variation in lifestyle, collection methods and detection platforms.^54,55^ To overcome this challenge, reverse metabolomics is performed at the repository scale to discover biomarkers consistent across multiple clinical studies. Here, a few metabolite-disease associations were detected, many of which were previously reported from independent studies. From our analysis, short and medium chain ACs were detected more frequently in individuals with diabetes relative to those with no disease reported, which supports previously published results (Figure 3a).^12,56–58^ Also in line with prior findings, some ACs were detected at higher rates in lung-related diseases and Chagas disease relative to controls, especially those with even-numbered chain lengths.^49,59,60^ Furthermore, our analysis revealed a previously unreported correlation between some short and medium even-chained ACs and osteoarthritis, based on a sample size of n = 1137 from multiple data sets.^61^ Specifically, C2:0, C6:0, C8:0, C10:0 and C10:1 ACs are detected in most samples from osteoarthritis patients across all cohorts in the repository, increasing confidence in this association.

**Figure 3:**
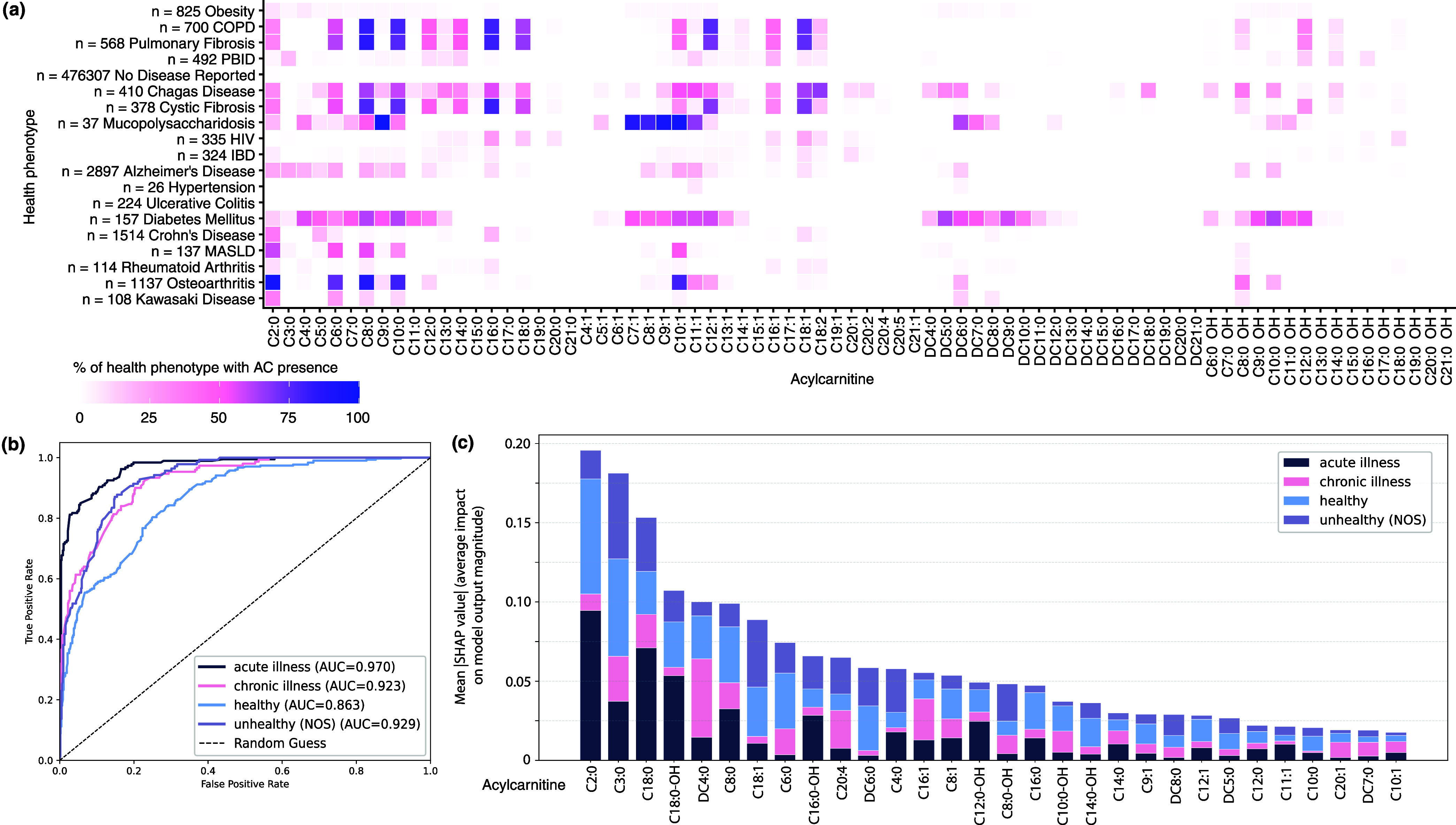
Using reverse metabolomics and machine learning to establish metabolite-disease relationships. (a) The heatmap displays the frequency of MS/MS matches across health phenotypes in humans reported in the PanReDU database with sample sizes provided. Abbreviations in figure are as follows: COPD is chronic obstructive pulmonary disease, PBID is primary bacterial infectious disease, HIV is human immunodeficiency virus infectious disease, IBD is inflammatory bowel disease and MASLD is metabolic dysfunction-associated steatotic liver disease. (b) Receiver operator characteristic (ROC) curves labeled by health status with area under the curve (AUC) values. (c) SHAP (SHapley Additive exPlanations) values for specific acylcarnitines driving unhealthy sample distinction in machine learning models.

Finally, we used machine learning to evaluate whether health phenotypes could be predicted using AC presence–absence profiles alone. Multiclass classification models were trained to distinguish among acute illness, chronic illness, healthy, and unhealthy (not otherwise specified) outcome categories using only binary AC detection features, without incorporating demographic or biological covariates. Across all evaluated model families, tree-based ensemble methods outperformed linear and probabilistic baselines, with Gradient Boosting achieving the strongest overall performance across accuracy, F1 scores and area-under-curve metrics (Figure 3b). Specifically, the Gradient Boosting model demonstrated robust discrimination across outcome classes, achieving high macro- and microaveraged AUROC and AUPRC values, indicating stable performance despite class imbalance and heterogeneity across data sets. To interpret the multiclass predictions, SHAP analysis was performed for the Gradient Boosting model. Feature importance analysis revealed that a relatively small subset of short- and medium-chain acylcarnitines, predominantly even-numbered species, contributed most strongly to model predictions across multiple health categories (Figure 3c). AC presence/absence patterns robustly distinguished between healthy and diseased individuals, but showed limited specificity across disease types. This observation may reflect the role of ACs as indicators of global mitochondrial and metabolic dysfunction, processes broadly perturbed across many pathological conditions rather than uniquely altered in a single disease state.^62^ Collectively, these results indicate that binary AC presence–absence patterns encode reproducible, interpretable metabolic signatures that differentiate overall health status at the repository scale, supporting their use as broad indicators of metabolic health rather than disease-specific diagnostic markers.

## Conclusion

This study represents a comprehensive analysis of ACs at the repository scale, examining where different structures are found in the body and relating them to human health outcomes. Our reverse metabolomics analysis detected ACs most frequently in blood and fecal samples, but found that they are ubiquitous throughout mammalian physiology. From this work, it is clear that ACs are nearly as prevalent in the GI tract as they are in blood, a finding that has not been presented before. Moreover, our analysis was able to establish metabolite-disease associations and recapitulate several previously published correlations. Overall, some acylcarnitines were detected markedly more frequently in samples from diseased individuals than those with no disease reported. Machine learning models validate AC profile as a potential biomarker platform with high accuracy but low specificity. Our combined results suggest acylcarnitines can serve as nonspecific health biomarkers beyond current screening protocols. Given existing clinical acylcarnitine detection infrastructure, these findings support the need to further examine acylcarnitine screening in adults.

## Supplementary Material





## Notes

Additional details about synthesis, LC-MS/MS collection and reverse metabolomics analysis (PDF)
USIs and MASST jobs for all synthesized acylcarnitines (ACs) (Table S1); presence-absence feature table showing where ACs are found in GNPS2 public data (Table S2); comparison of MS/MS spectral matches from crude vs pure ACs (Table S3); comparison of MS/MS spectral matches between isomers (Table S4); multiclass classification performance using acylcarnitine presence–absence features only (Table S5); overview of PanReDU database sample sizes and MS/MS search results (XLSX) Code (Table S6); data tables and other Supporting Information can be found at https://github.com/keysaj11/Acylcarnitine-Paper. USIs and MASST Task IDs associated with each synthesized acylcarnitine are found in Table S1; the spectral library (https://gnps2.org/status?task=6476c66df8a1470bbc9c2823f19d4417) is deposited in and can be downloaded from GNPS public MS/MS libraries (XLSX)
