# Supplementary material for: Exploring Acylcarnitine Metabolism Using Reverse Metabolomics
Source: Anal Chem. 2026 Jun 4;98(23):17217–25. doi: 10.1021/acs.analchem.6c01418 (PMC13276854; doi:10.1021/acs.analchem.6c01418)
Supplement: Supplementary file 1 [file ac6c01418_si_001.pdf]

Supporting Information

Exploring Acylcarnitine Metabolism Using Reverse Metabolomics

AUTHOR NAMES

Allison J. Keys[1], Daniel J. Wiener[1], Sara M. Pacini[1], Chengze Sun[3], Lindsay E. Sandusky[1], Huaiyang Zhong[2], Emily C. Gentry[1]*

AUTHOR ADDRESS

[1] Virginia Tech, Department of Chemistry, Blacksburg, VA, 24061 USA
[2] Virginia Tech, Grado Department of Industrial and Systems Engineering, Blacksburg, VA, 24061, USA
[3] Hong Kong University of Science and Technology (HKUST), Department of Electrical Engineering, Hong Kong
* correspondence to egentry@vt.edu

Table of Contents

S1. Synthesis of Acylcarnitine……………………………………………………………...……S3
S1.1. Materials……………………………………………………………………………S3
S1.2. Saturated and Unsaturated Acylcarnitines…....………………....………………....S3
S1.3. Dicarboxylated Acylcarnitine……………………………………………………..…S3
S1.4. Hydroxylated Acylcarnitine………………………………………………………..S3
S2. Reverse Metabolomics Workflow……………………………………………………...S4
S2.1. Optimization of LC-MS/MS for Hydroxylated ACs……………………………..S4
S2.2. Effects of Regiochemistry for Hydroxylated Fatty Acids……………………………S4
S2.3. Samples in PanReDU Database……………………………………………………S4
Figure S1……………………………………………………………………………………..S5
Figure S2……………………………………………………………………………….…S6
Figure S3……………………………………………………………………………………..S7
Figure S4……………………………………………………………………..…………....S8
Figure S5…………………………………………………………………….……….……S9
Figure S6………………………………………………………………….………..……..S10

S1. Synthesis of Acylcarnitines

S1.1. Materials

Organic solutions were concentrated under reduced pressure on a Büchi rotavapor R-205 using a Büchi Heating Bath B-490. All reactions were conducted in a well-ventilated fume hood. L-carnitine was purchased from Ambeed. Dry dimethylformamide (DMF) and 3,4-dihydropyran were purchased from Thermo Scientific. Tert-butylchlorodimethylsilane was purchased from Combi-Blocks. 4-(dimethylamino)pyridine (DMAP) was purchased from Novabiochem. 1-Ethyl-3-(3'-dimethylaminopropyl)carbodiimide (EDC·HCl) was purchased from Matrix Scientific. All fatty acid suppliers can be found in **Table S1**.

S1.2. Saturated and Unsaturated Acylcarnitines

The respective fatty acids (1 equiv, 87μmol), DMAP (1.2 equiv, 100 μmol) and EDC·HCl (1.2 equiv, 100 μmol) were added to a reaction vessel with 400 μL dimethyl formamide (DMF). This solution was allowed to stir at room temperature for 15 mins, then L-carnitine (1.1 equiv, 96 μmol) was added. This reaction mixture was stirred overnight at room temperature. C21:1 acylcarnitine was run at half scale due to limited starting material.

*S1.3. Dicarboxylated Acylcarnitine*s

A similar protocol was followed for dicarboxylated acylcarnitines, where the respective fatty acid (1 equiv, 0.1 mmol), DMAP (1.2 equiv, 0.12 mmol) and EDC·HCl (1.2 equiv, 0.12 mmol) were added to a reaction vessel followed by 500 μL dry DMF. This solution was allowed to stir for 15 mins, then carnitine (1.2 equiv, 0.12 mmol) was added. This reaction mixture was stirred overnight at room temperature.

S1.4. Hydroxylated Acylcarnitines

For hydroxylated ACs, the synthetic method was adapted from the procedure described above. In this protocol, tert-butylchlorodimethylsilane (1.2 equiv, 100 μmol) was added to the hydroxylated fatty acid (1 equiv, 87 μmol) in 400 μL DMF to protect the alcohol. This in situ protection reaction was allowed to stir for 15 minutes before addition of DMAP (1.2 equiv, 100 μmol) and EDC·HCl (1.2 equiv, 100 μmol). The resulting solution was allowed to stir for 15 mins, then L-carnitine (1.1 equiv, 96 μmol) was added. This reaction mixture was stirred overnight at room temperature. No deprotection was performed on the reaction mixture, as the *tert*-Butyldimethylsilyl (TBS) group dissociates during LC-MS analysis.

For terminal hydroxylated acylcarnitines with chain lengths between C18-C21, the protocol was modified. 3,4-Dihydropyran (1.5 equiv, 71.4 μmol) was added to the terminally hydroxylated fatty acid (1 equiv, 47.6 μmol) in 300 μL DMF to protect the alcohol and this reaction was allowed to stir for 15 minutes before addition of DMAP (2 equiv, 95.2 μmol) and EDC·HCl (1.5 equiv, 71.4 μmol). The resulting solution was stirred for an additional 15 mins, wherein L-carnitine (1.1 equiv, 52.3 μmol) was added. This reaction mixture was stirred overnight at room temperature.

S2. Reverse Metabolomics Workflow

S2.1. Optimization of LC-MS/MS for Hydroxylated ACs

There is the possibility to form regioisomers with the same $[M]^+$ peak for hydroxylated ACs. To control for this, only spectra that contain the distinguishing fragments of an acylcarnitine (m/z of 60.0808 and 85.0284) were selected for the reverse metabolomics workflow (**Figures S2** and **S4**). The desired acylcarnitine eluted before the regioisomer, and while some of the longer-chain ACs (C18-21) eluted at the same time as the regioisomer, LC optimization was performed to provide separation. The LC-MS/MS method for these long chain hydroxylated ACs used the same general parameters as the other ACs, but with a different eluent gradient. The following gradient was used: 0 – 1.5 min 5% B, 1.5 – 3.0 min 70% B, 3.0 – 10.0 min 100% B, 10.0 – 11.5 min 100% B, 11.5 – 11.6 min 5% B, 11.6 – 13.0 min 5% B.

S2.2. Effects of Regiochemistry for Hydroxylated Fatty Acids

Once it was determined that both the internal and external hydroxylated gave similar spectra and matches in GNPS2 (**Table S4**), we chose to move forward with the terminal hydroxylated fatty acid for use in synthesis. This was done first because of the higher commercial availability of the terminal hydroxylated fatty acids. Next, the terminal hydroxylated AC has no stereochemistry about the hydroxyl group, and therefore the acylcarnitine produced does not possess a diastereomer. This can clearly be seen in the extracted ion chromatography (XIC) of both the terminal hydroxylated C6 (**Figure S4a**) and the internal hydroxylated C6 (**Figure S4b**). Finally, the peak area ratio of the acylcarnitine compared to the regioisomer was almost twice as large for the terminal as compared to the internal hydroxylated acylcarnitine. The peak area of the internal hydroxylated AC was approximately, on average, 40% of the regioisomers peak area. The peak area of the terminal hydroxylated AC was approximately, on average, 80% of the regioisomers peak area. This concluded that the terminal hydroxylated fatty acid is the better choice for synthesis, and what was used for data processing. Also shown in **Figure S4c and 4d** is the MS/MS spectra for both the acylcarnitine and regioisomer of a C3 hydroxylated C6 and the structure of pertinent fragments.

S2.3. Samples in PanReDU Database

Statistics from the data files in PanReDU (966,340) and our matches from GNPS2 with PanReDU data (37,690) can be found in **Table S6** and includes tissue, sample type and species data. Categories are merged according to code for **Figure 2c, Figure 3a** and **Figure S5** that can be found on GitHub. Our analysis illustrates that within PanReDU, approximately 57% of breast milk data files, 68% of mucosa data files and 37% of biliary system data files contain acylcarnitines. However, most of our AC matches within tissue type come from blood (8,195). Additionally, while most of our GNPS2 matches in PanReDU come from the animal sample type (33,031), approximately 16% of food/beverage sample types contain acylcarnitines while only 5% of animal samples contain ACs. Finally, while most matches in animals are from humans (18,759), only approximately 4% of human data files contain ACs while approximately 13% of mice data files (11,913) contain acylcarnitines.

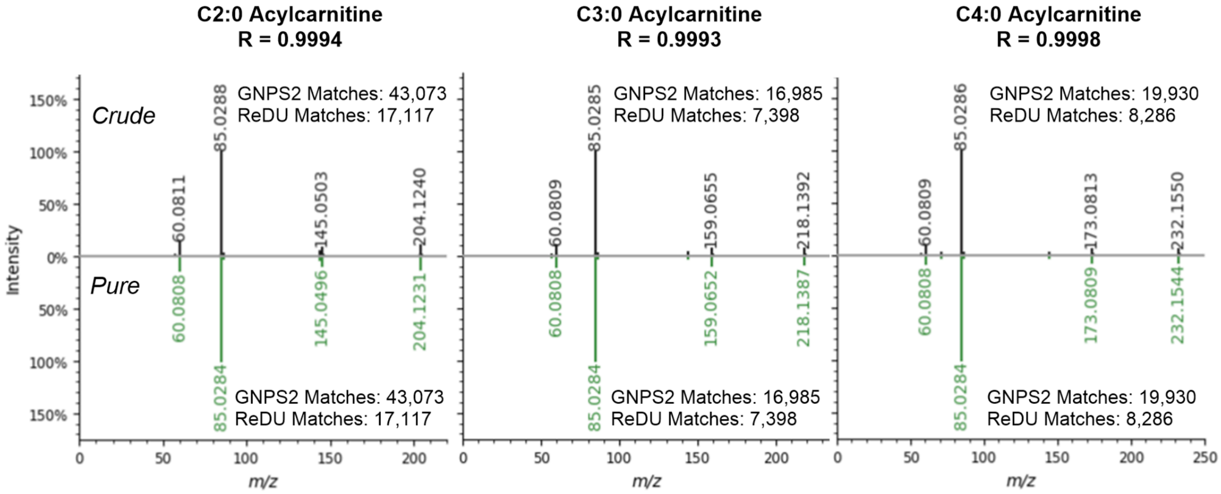

Figure S1. MS/MS mirror plots for crude vs. pure acylcarnitines. MS/MS spectra detected from crude reaction mixtures have cosine similarity scores of 0.999 when compared to pure acylcarnitines from commercial sources.

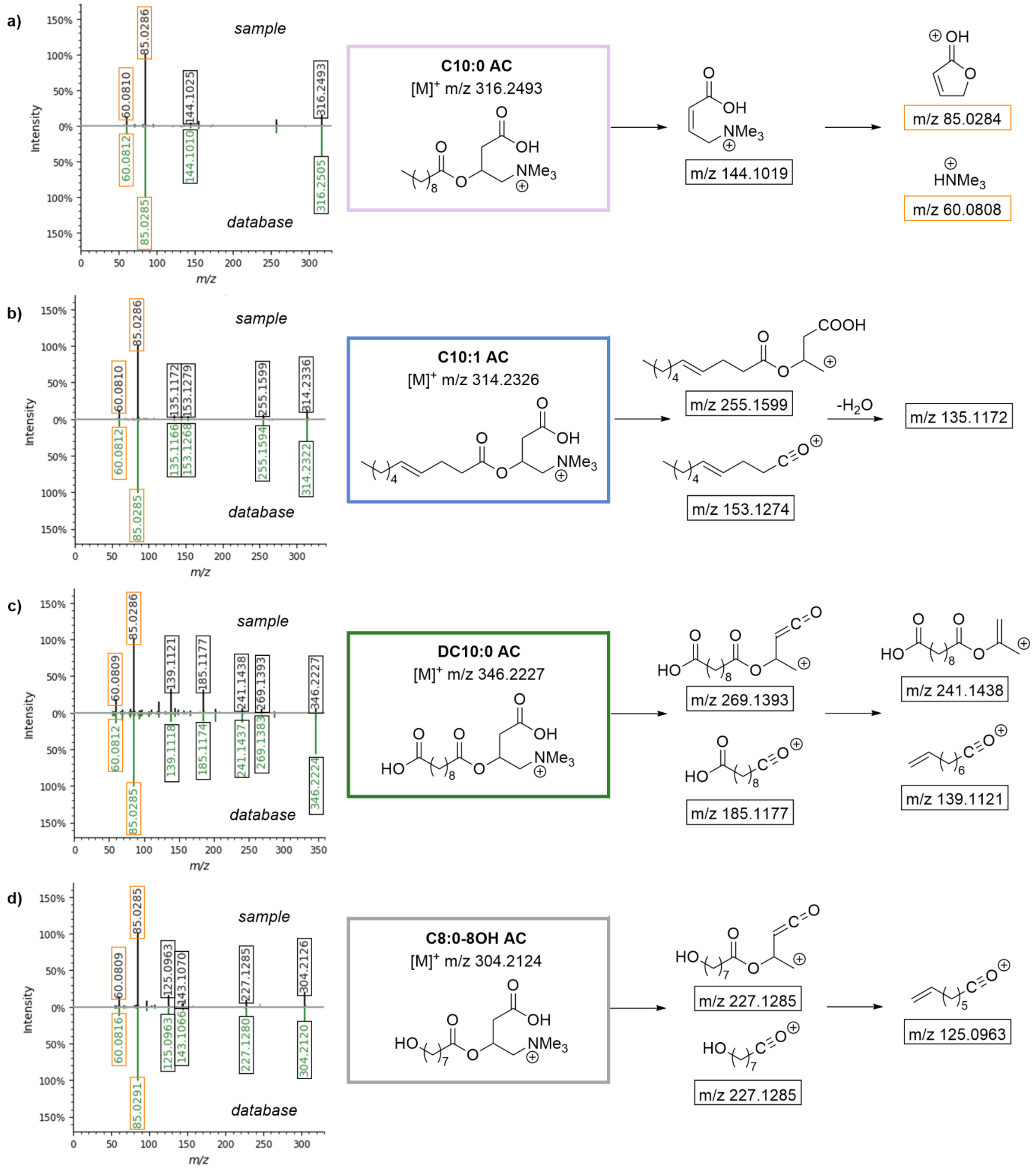

Figure S2. Representative MS/MS fragmentation used for quality control of synthesized **a)** saturated **b)** unsaturated **c)** dicarboxylated **d)** hydroxylated acylcarnitines.

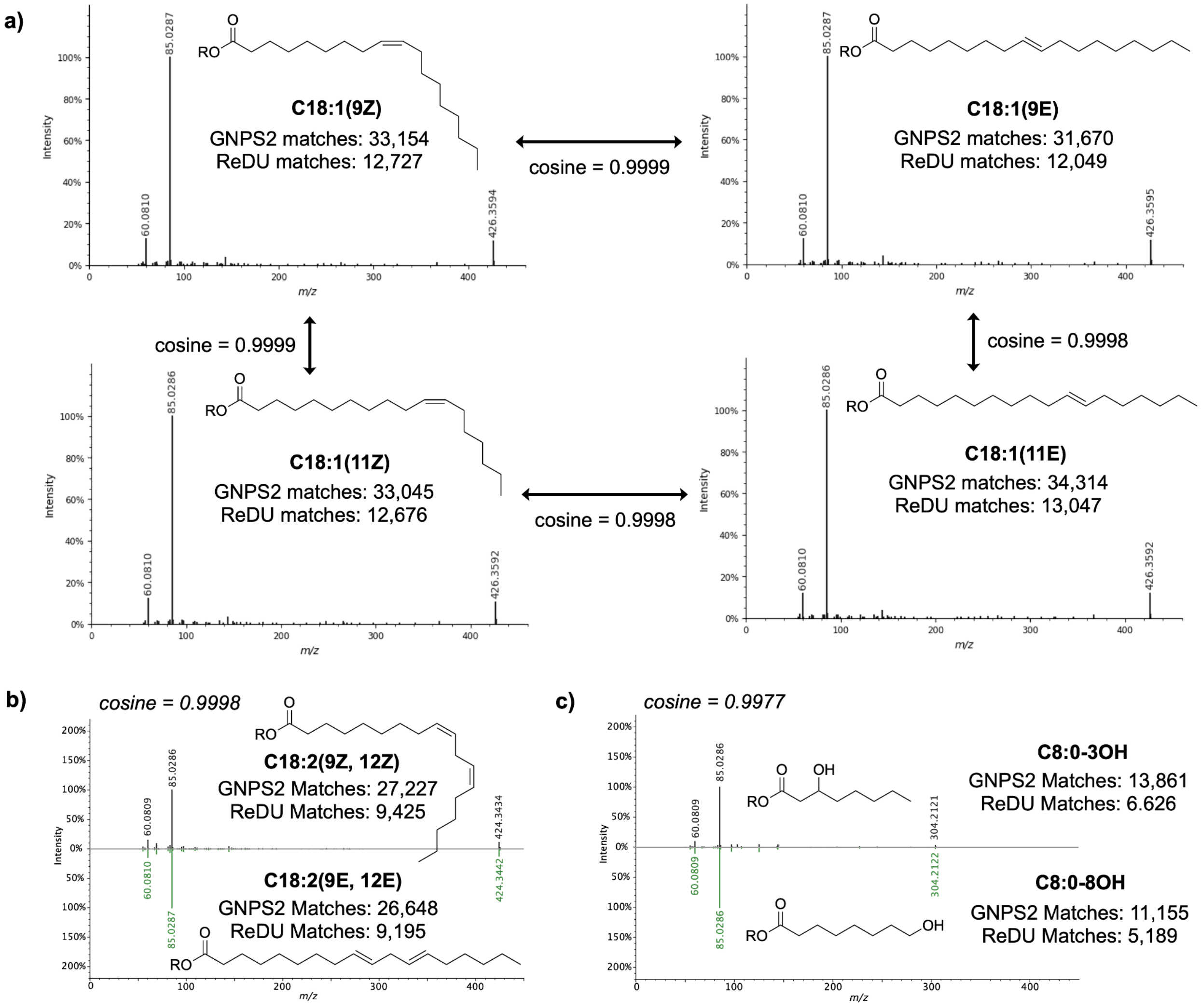

Figure S3. MS/MS spectra of different acylcarnitines and their respective matches in GNPS2 and PanReDU. **a)** Comparison of singly unsaturated C18 acylcarnitines with *cis*- or *trans*-versions of the alkene at the C9 and C11 positions. **b)** Doubly unsaturated C18 acylcarnitines with *cis*- or *trans*- alkenes at the C9 and C12 positions. **c)** Hydroxylated C8 acylcarnitines where the hydroxyl group is present at the C3 or C8 positions.

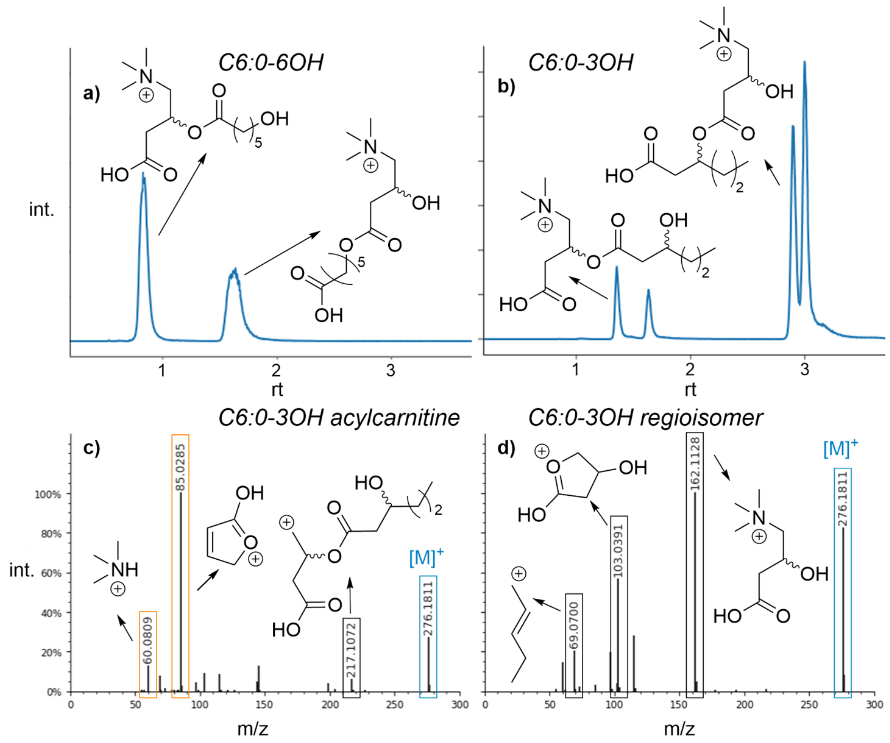

Figure S4. XIC of 276.1806 C6 hydroxylated fatty acid showing distinct regioisomers. **a)** The terminally hydroxylated C6, with two distinct peaks, the acylcarnitine on the left and the regioisomer on the left. **b)** The C3 hydroxylated C6, with four distinct peaks. The pair of peaks on the left contains the diastereomers of the acylcarnitine and the right pair of peaks contain the diastereomers of the regioisomer. **c)** The MS/MS spectra of the C3 hydroxylated C6 acylcarnitine and its distinct peaks and the associated structures. **d)** The MS/MS spectra of the C3 hydroxylated C6 regioisomer and its distinct peaks and the associated structures.

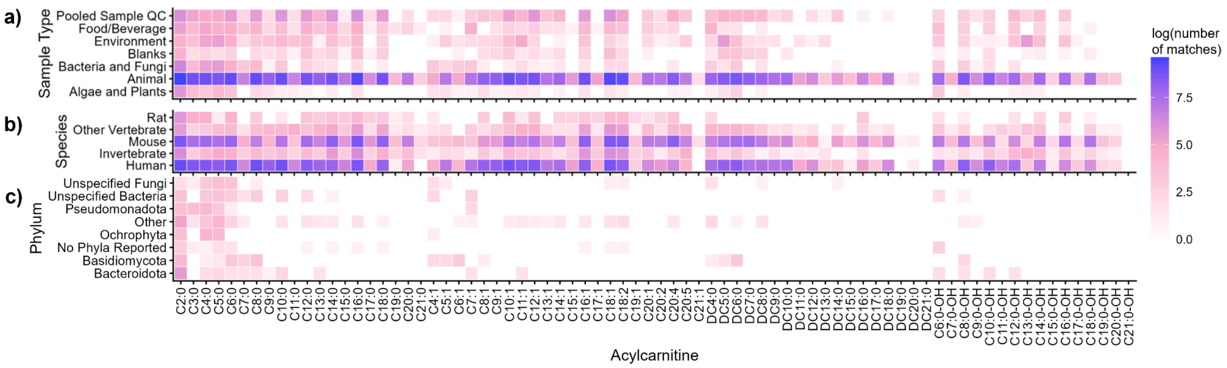

Figure S5. Heatmaps showing the number of MS/MS matches across sample types. Values are logarithmic with the scale shown on the right. **a)** MS/MS matches across all sample types within PanReDU aggregated by the “SampleType” category. **b)** Number of matches across different species when filtered to “animal” samples in the “SampleType” category. All species names in “NCBITaxonomy” were transformed to their common names and the top three most populated species are named; mouse, human and rat. All other species were merged into “Other Vertebrate” or “Invertabrate”. **c)** Few MASST results were detected in bacterial and fungal samples, shown by phyla. These results were filtered to show only MS/MS matches to bacterial and fungal samples.

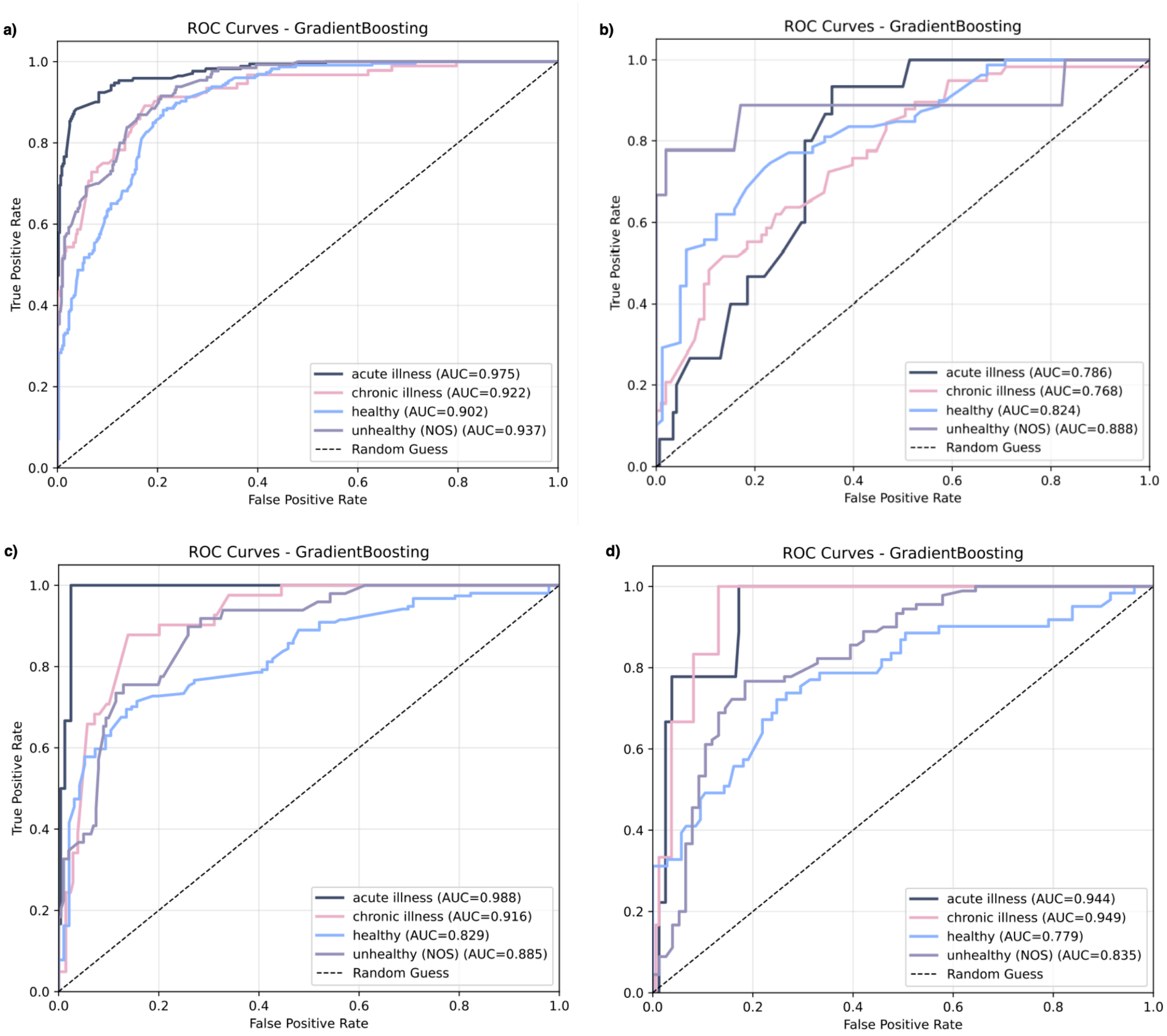

Figure S6. Machine learning model performance in a single instrument platform and sample type. To verify performance in the absence of confounding factors, four data subsets were retrained and tested: a) Thermo QExactive data, b) Bruker Maxis Q-ToF data, c) fecal samples and d) blood samples.
